# The Local Environment of Loop Switch 1 Modulates the Rate of ATP-Induced Dissociation of Human Cardiac Actomyosin

**DOI:** 10.3390/ijms23031220

**Published:** 2022-01-22

**Authors:** Akhil Gargey, Yuri E. Nesmelov

**Affiliations:** 1 Department of Physics and Optical Science, University of North Carolina at Charlotte, Charlotte, NC 28223, USA; airagava@uncc.edu; 2 Department of Biological Science, University of North Carolina at Charlotte, Charlotte, NC 28223, USA

**Keywords:** human cardiac myosin, loop switch 1, loop switch 2, P-loop, ATP, ADP

## Abstract

Two isoforms of human cardiac myosin, alpha and beta, share significant sequence similarities but show different kinetics. The alpha isoform is a faster motor; it spends less time being strongly bound to actin during the actomyosin cycle. With alpha isoform, actomyosin dissociates faster upon ATP binding, and the affinity of ADP to actomyosin is weaker. One can suggest that the isoform-specific actomyosin kinetics is regulated at the nucleotide binding site of human cardiac myosin. Myosin is a P-loop ATPase; the nucleotide-binding site consists of P-loop and loops switch 1 and 2. All three loops position MgATP for successful hydrolysis. Loops sequence is conserved in both myosin isoforms, and we hypothesize that the isoform-specific structural element near the active site regulates the rate of nucleotide binding and release. Previously we ran molecular dynamics simulations and found that loop S291-E317 near loop switch 1 is more compact and exhibits larger fluctuations of the position of amino acid residues in beta isoform than in alpha. In alpha isoform, the loop forms a salt bridge with loop switch 1, the bridge is not present in beta isoform. Two isoleucines I303 and I313 of loop S291-E317 are replaced with valines in alpha isoform. We introduced a double mutation I303V:I313V in beta isoform background and studied how the mutation affects the rate of ATP binding and ADP dissociation from actomyosin. We found that ATP-induced actomyosin dissociation occurs faster in the mutant, but the rate of ADP release remains the same as in the wild-type beta isoform. Due to the proximity of loop S291-E317 and loop switch 1, a faster rate of ATP-induced actomyosin dissociation indicates that loop S291-E317 affects structural dynamics of loop switch 1, and that loop switch 1 controls ATP binding to the active site. A similar rate of ADP dissociation from actomyosin in the mutant and wild-type myosin constructs indicates that loop switch 1 does not control ADP release from actomyosin.

## 1. Introduction

**Figure 1:**
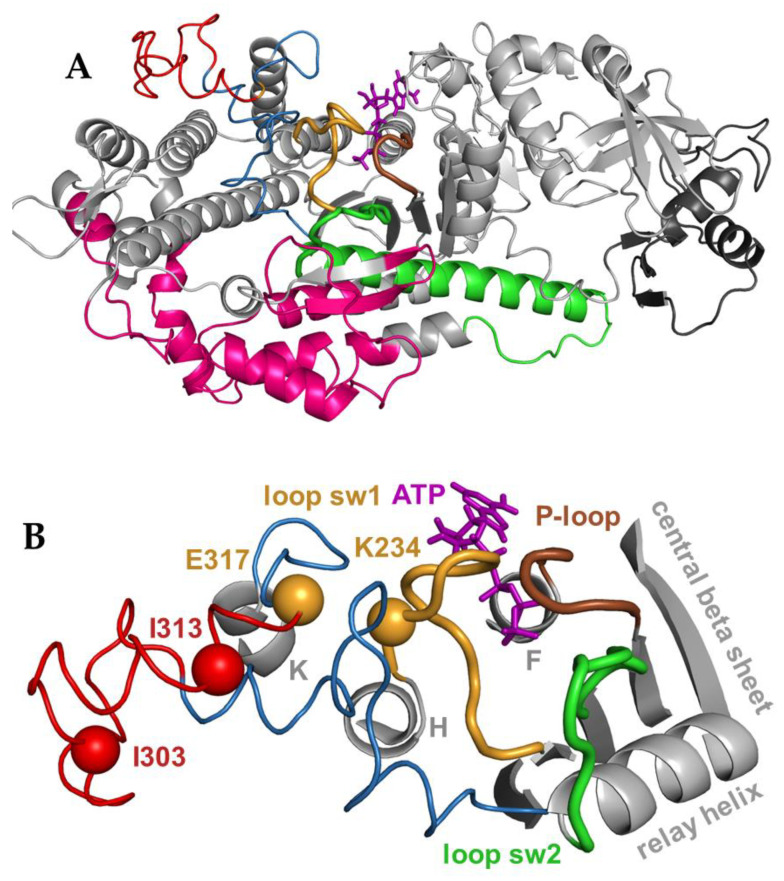
(**A**) Myosin head, location of the active site and loop S291-E317. Bulk colored regions and elements, pink, the actin binding interface, green, the relay helix and loop, dark grey, the converter domain. Colored loops show the myosin active site. (**B**) Myosin active site, formed by P-loop (brown), loop switch 1 (orange), and loop switch 2 (green). Beta isoform of human cardiac myosin, 4db1.pdb. ATP bound to the active site is colored purple. Loop L268-D324 is colored blue, and the loop S291-E317 part of it, discussed in the paper, is colored red. Residues I303 and I313 are shown as red spheres. Residues E317 and K234 are shown as orange spheres. K, H, F, and relay are helices connected to the C-termini of loops L268-D324, switch 1, P, and switch 2, respectively. N-termini of all mentioned loops are connected to strands 4–7 of the central beta sheet. In this work, we replaced residues I303 and I313 with valines, as they are in alpha isoform. According to the computer simulations, residues E317 and K234 form salt bridge in alpha isoform (populated 35.2% during the calculated molecular dynamics trajectory, not populated in beta isoform). Loop L268-D324 is shown without helical content to simplify the image.

Myosins perform various motor functions in living organisms, regardless of high conservation of the core sequence and structure of the motor domain in all myosin classes [1,2]. One can speculate that the fold of the myosin head determines the general function of the molecule, and the kinetic properties of different myosins are determined by the sequence-specific residue–residue interactions. Previously we hypothesized that electrostatic interactions within the myosin head modulate kinetic steps of the actomyosin cycle. To examine the hypothesis, we chose two isoforms of human cardiac myosin, alpha and beta (genes *MYH6* and *MYH7*), which have 93% sequence identity in the motor domain. Despite the high sequence conservation, actomyosin with alpha isoform binds ATP faster and has a lower affinity to ADP than beta isoform, which makes alpha isoform a faster motor. Previously, based on the available crystal structure of the motor domain of beta isoform, we built a homology model of alpha isoform and ran molecular dynamics simulations for both isoforms. We compared the fluctuation of the position of amino acid residues within structural elements of the myosin head and assessed permanent, isoform-specific salt bridges [3]. We found such bridges in several parts of the myosin head domain. We showed that electrostatic interactions in the SH1:SH2 region of the myosin head [4] and the salt bridge between loop 1 and myosin backbone [5] modulate the kinetics of nucleotide binding and release from human cardiac actomyosin. Both loop 1 and the SH1:SH2 region are located relatively far from the active site of the myosin head, but they are connected to the loops of the active site. Three loops form the myosin active site, P-loop, and loops switch 1 and 2 (Figure 1). All three loops are connected to strands 5, 6, and 4 of the central beta sheet (strands notation from [6]). Other ends of the loops of the active site are connected to helices, linking the loops to different regions of the myosin head. Thus, loop switch 1 is connected to helix H, and the P-loop is connected to helix F, and loop 1 links other sides of helices H and F. As we previously found, the removal of the salt bridge, linking loop 1 and myosin backbone, increases the rate of ATP binding to actomyosin, probably due to the increased dynamics of loop 1. Removal of the salt bridge does not change the rate of ADP release. The effect of perturbed electrostatic interactions in the SH1:SH2 region apparently communicates to the active site via relay helix, which is the continuation of loop switch 2. Changed electrostatic interactions within the SH1:SH2 region increase the rate of ADP release and do not affect the rate of ATP binding to actomyosin. Therefore, our previous findings suggest that in human cardiac actomyosin, loop switch 1 regulates the rate of ATP binding to actomyosin, and loop switch 2 regulates the rate of ADP release.

We report the results of our study of the effect of the isoform-specific loop S291-E317 on the kinetics of ATP binding and ADP release from human cardiac actomyosin. Loop S291-E317 is the part of the larger loop L268-D324 which connects strand 7 of the central beta sheet and helix K, and is located near loop switch 1. Trajectory analysis of our molecular dynamics simulations showed that loop S291-E317 exhibits the isoform-specific difference of fluctuation of the position of amino acid residues. Loop S291-E317 contains residues I303 and I313 in beta isoform, which are replaced with valines in alpha isoform. The loop has increased helical content in beta isoform. Trajectory analysis showed the formation of salt bridge E317:K234 with loop switch 1 in alpha isoform, the bridge is populated during 35.2% of the time of the trajectory. We hypothesize that such differences in residue dynamics and electrostatic interaction of loop S291-E317 with loop switch 1 may contribute to the difference in kinetic steps in alpha and beta isoforms of human cardiac actomyosin. We also hypothesize that because of the proximity to loop switch 1, isoform-specific loop S291-E317 affects only ATP binding and not ADP release. To test our hypotheses, we introduced double mutation I303V:I313V in beta isoform background to produce alpha-isoform specific loop S291-E317, and eventually, alpha-isoform specific salt bridge E317:K234. We characterized the kinetics of ATP-induced actomyosin dissociation and ADP release in the wild-type (WT) construct and in the mutant, using stopped-flow spectrofluorometry. We found a faster rate of ATP-induced actomyosin dissociation and a similar rate of ADP release in the mutant. We conclude that in human cardiac actomyosin, loop switch 1 most likely controls the rate of ATP binding and not ADP release from the active site after ATP hydrolysis.

## 2. Results

### 2.1. The Rates of ATP Binding and Actomyosin Dissociation Are Affected by the Mutation

**Figure 2:**
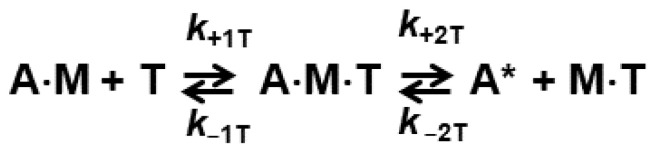
ATP-induced actomyosin dissociation. A = pyrene labeled actin, M = myosin, T = ATP. A* = actin with the high pyrene fluorescence.

**Figure 3:**
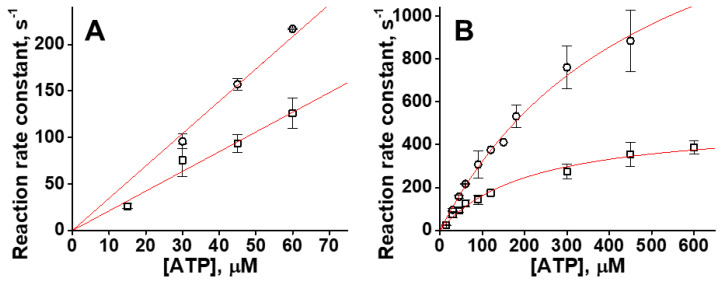
ATP-induced actomyosin dissociation. Squares, WT, data from [4], *N* = 3. Circles, mutant, *N* = 3. (**A**). Observed reaction rates at low [ATP], fitted with a straight line, the second-order reaction rate constant is determined from the slope of the line. WT and mutant, respectively, *K*_1T_*k*_+2T_ = 2.12 ± 0.21 μM^−1^s^−1^ and 3.54 ± 0.07 μM^−1^s^−1^, *p* = 0.0029. (**B**). Reaction rates fitted with a hyperbola. Rates *k*_+2T_ = 491.5 ± 74.1 s^−1^ and 1909.0 ± 354.8 s^−1^, *p* = 0.0168, for WT and mutant, respectively. Equilibrium dissociation constant 1/*K*_1T_ = 214.2 ± 24.9 μM and 506.2 ± 161.7 μM, *p* = 0.0738. Data points are mean ± SD. *N* is the number of biological replicates.

We used pyrene-labeled actin as an indicator of ATP-induced actomyosin dissociation. When actin strongly bound to myosin, pyrene fluorescence is quenched. Actomyosin dissociation results in the increased fluorescence of pyrene labeled actin. In the case of the WT myosin construct the maximum rate of actomyosin dissociation was achieved at 450 µM–900 µM ATP. In the case of the I303V:I313V mutant, kinetics of actomyosin dissociation was significantly faster. At the ATP concentration of 150 µM and higher, the obtained transients were mostly flat, indicating that the majority of actomyosin dissociation occurs during the dead time of the stopped flow fluorometer. Due to this fast kinetics, the error of the kinetic rate determination rises significantly at high ATP concentrations. Assuming rapid equilibrium of the ATP-actomyosin collision complex formation (step 1 in Figure 2), we fitted obtained transients with a single-exponential function to determine reaction rates of actomyosin dissociation at different concentrations of ATP. The obtained rates were fitted with a hyperbola to determine *k*_+2T_ and 1/*K*_1T_ (Figure 3), obtained rate constants are *k*_+2T_ = 491.5 ± 74.1 s^−1^ (WT) and *k*_+2T_ = 1909.0 ± 354.8 s^−1^ (mutant), *p* = 0.0168. The equilibrium constant of ATP-actomyosin collision complex dissociation 1/*K*_1T_ = 214.2 ± 24.9 μM (WT) and 506.2 ± 161.7 μM (mutant), *p* = 0.0738. The rate constant *K*_1T_*k*_+2T_ was determined directly from the slope of the linear fit of the dependence of reaction rates on ATP concentration (Figure 3), *K*_1T_*k*_+2T_ = 2.12 ± 0.21 μM^−1^s^−1^ (WT) and 3.54 ± 0.07 μM^−1^s^−1^ (mutant), *p* = 0.0029, Table 1. Since the rate constant *k*_+2T_ for the mutant is high, the error of the rate determination is high, and we fitted obtained transients with the numerical solution of the system of differential equations corresponding to the reaction scheme of ATP-induced actomyosin dissociation (Figure 2). Results of the fit to the numerical solution of differential equations are presented in Table 1, except the data for *K*_1T_*k*_+2T_, obtained directly from the fit shown in Figure 3. For the WT actomyosin, both fits produced similar values for the rate constant *k*_+2T_, 491.5 ± 74.1 s^−1^ for the fit with a hyperbola, and 406.2 ± 14.1 s^−1^ for the fit with differential equations. The equilibrium dissociation constant of the ATP-actomyosin collision complex determined from the hyperbola fit was 1/*K*_1T_ = 214.2 ± 24.9 μM, the same constant determined from the fitted parameters of the solution of the differential equations *k*_−1T_/*k*_+1T_ was smaller, 89.6 ± 6.5 μM, showing that the solution of the differential equations reveals tighter binding of ATP and actomyosin in the collision complex. Data for the WT actomyosin were reported in our previous study [4]. For the mutant actomyosin, fits with hyperbola and with differential equations produced similar results, *k*_+2T_ = 1909.0 ± 354.8 s^−1^ and 2307.6 ± 389.6 s^−1^, equilibrium dissociation constant (1/*K*_1T_) = 506.2 ± 161.7 μM and 461.7 ± 86.7 μM, respectively. Among rate constants obtained from fits with differential equations, the rate constants *k*_−2T_ are statistically similar for the WT and mutant, *k*_−2T_ = 3.6 ± 2.5 s^−1^ (WT) and 0.9 ± 0.5 s^−1^ (mutant), *p* = 0.3713 (Table 1). All other rate constants are different for the WT and mutant actomyosin (Table 1), *k*_+1T_ = 7.42 ± 1.35 μM^−1^s^−1^ (WT) and 12.83 ± 1.61 μM^−1^s^−1^ (mutant), *p* = 0.0348, *k*_−1T_ = 661.0 ± 72.9 s^−1^ (WT) and 5877.84 ± 1025.1 s^−1^ (mutant), *p* = 0.01214, *k*_+2T_ = 406.2 ± 14.1 s^−1^ (WT) and 2307.6 ± 389.6 s^−1^ (mutant), *p* = 0.0136. The high value of the standard deviation of the rate constant *k*_−1T_ reflects shallow χ-squared surface for this parameter in our fits. The rate constants obtained from our experiments clearly show an increased rate of ATP-induced actomyosin dissociation in the mutant. The rate constants *k*_+2T_ for the WT and mutant are statistically different, and our data show that I303V:I313V mutation leads to the increased rate of ATP-induced actomyosin dissociation.

### 2.2. The Rate of ADP Release from Actomyosin Is Not Affected by the Mutation

We monitor the release of ADP from actomyosin in the transient experiment when we rapidly mix actomyosin with the premixed ATP and ADP. The concentration of ATP kept constant in the experiment (600 µM), and we vary concentration of ADP in the mixture from 10 µM to 200 µM. For both myosin constructs, the WT and the mutant, we observed transients, which are best fitted with a two-exponential function. This observation confirms that ADP and actomyosin are not in fast equilibrium, as for example in the case of rabbit skeletal myosin. Therefore, the kinetics of the ADP inhibited ATP-induced dissociation of actomyosin can be modeled as two parallel reactions, actomyosin interacting with ADP, and actomyosin interacting with ATP. In the model, the binding of ATP results in the irreversible actomyosin dissociation. The binding of ADP slows down the reaction because the ATP-induced actomyosin dissociation is possible only after ADP release for actomyosin. Our experiments show that in the case of human cardiac myosin, mixing actomyosin with ADP does not result in actomyosin dissociation. Our two-exponential fits show gradual increase in the slow component with an increase in ADP concentration. The slow component reaches approximately 90% of the transient when [ADP] = 200 µM. Considering ATP concentration 600 µM in the experiment, one can suggest that ADP binds actomyosin faster than ATP. We fitted transients, obtained in this experiment to the solution of the differential equations, corresponding to the reaction scheme of two parallel reactions, and did not assume the fast equilibrium of actomyosin and ADP upon rapid mixing. Traces obtained at different concentrations of ADP were fitted simultaneously to determine the reaction rate constants (Table 1). We found that ADP binds the mutant actomyosin faster than the WT, *k*_+D_ = 26.6 ± 8.1 µM^−1^s^−1^ (WT) and 41.6 ± 3.1 µM^−1^s^−1^ (mutant), *p* = 0.02724, that correlates with the faster binding of ATP. The rate constants of ADP dissociation are statistically similar, *k*_−D_ = 199.6 ± 51.8 s^−1^ (WT) and 175.7 ± 8.7 s^−1^ (mutant), *p* = 0.8822, (Table 1). While we see the faster binding of ADP to mutant actomyosin, our data show that I303V:I313V mutation does not affect the rate of ADP dissociation from actomyosin.

## 3. Discussion

Our data directly show that actomyosin dissociation step *K*_1T_*k*_+2T_ is affected by the mutation. These data are consistent with an increase in the rate constant of actin dissociation *k*_+2T_. Regardless of the error of determination of the rate constant *k*_−1T_ for the mutant, the calculated equilibrium constants of the collision complex dissociation 1/*K*_1T_ are statistically different. ATP binds the active site faster than in the WT, but not as fast as the diffusion-controlled limit [4], and we conclude that the significant amount of time the active site is closed or is in non-favorable conformation for ATP binding. The increased rate of the collision complex dissociation *k*_−1T_ is compensated by the increased rate of actomyosin dissociation *k*_+2T_. These rates are the same order of magnitude, ensuring that the reaction of ATP binding is a two-step reaction. Our modeling of transients of the mutant using numerical solutions of the corresponding system of differential equations showed that increase or decrease in the rate constant *k*_+1T_ results in a small variation in the rate *k*_+2T_, but in a large variation in the rate *k*_−1T_. Setting *k*_+1T_ of the mutant to the value for the WT actomyosin reduces the quality of the fit and results in the value of the rate *k*_−1T_ being smaller than the rate *k*_+2T_, which should make the reaction a one-step reaction, not observed experimentally.

Three conserved loops form the myosin active site, P-loop, and loops switch 1 and switch 2. Loop switch 1 has the consensus sequence NxxSSR, and mutagenesis studies of conserved residues N238, S242, and R243 (sequence of human cardiac myosin, beta isoform) confirmed the importance of these residues for holding nucleotide in the active site and ATP hydrolysis [7,8,9,10,11]. Missense mutation of R243 in unconventional myosin VIIa results in deafness [12]. Conformational change of loop switch 1 upon ATP binding (loop closure) is directly coupled to actomyosin dissociation [13,14]. Coupling of the loop opening and actomyosin conformational changes is currently less understood. There are two hypotheses on the role of loop switch 1 opening in actomyosin kinetics. One is that the loop controls the release of abstracted phosphate, and therefore the binding of myosin and actin [15]. Another hypothesis is that the loop controls ADP release from the active site [16]. The difference is significant: control of phosphate release affects the entrance into the force-generating strongly bound state of actomyosin. Control of ADP release determines the duration of the strongly bound state, and therefore the force produced by the muscle. Our recent studies indicate that perturbation of loop 1, connected to loop switch 1 by helix H, resulted in modulation of the rate of nucleotide binding and actomyosin dissociation, or loop switch 1 closure. The rate of ADP release was not affected by perturbation of loop 1. Perturbation of the electrostatic interactions in the SH1-SH2 region caused an increased rate of ADP release and did not change the kinetics of ATP binding and actomyosin dissociation. These findings indicate that conformational changes in loops switch 1 and switch 2 are related to different events within the myosin head, either structural changes at the actin binding interface or conformational changes within the SH1-SH2 region, resulting in the recovery stroke and power stroke. In our current work, we studied how the local environment of loop switch 1 affects actomyosin interaction, and our results support the hypothesis that loop switch 1 controls nucleotide binding and does not control ADP release from actomyosin. It is interesting to mention that according to our previous molecular dynamics simulations, loop S291-E317 shows a smaller fluctuation of residues in alpha isoform than in beta isoform, and trajectory analysis showed the formation of salt bridge E317:K234 between loop S291-E317 and loop switch 1. These suggest that mutation I303V:I313V, introduced in beta isoform, should not increase the flexibility of loop switch 1. Most likely, the mutation produces a small conformational change near loop switch 1, thus changing loop switch 1 structural dynamics. We understand that the environment of loop 1 might be myosin-specific, and our findings may apply solely to human cardiac myosin.

## 4. Materials and Methods

### 4.1. Reagents

*N*-(1-pyrene)iodoacetamide (pyrene) was purchased from Life Technologies Corporation (Grand Island, NY, USA). ATP, ADP, and phalloidin were purchased from Sigma-Aldrich (Milwaukee, WI, USA). All other chemicals were purchased from Fisher Scientific (Waltham, MA, USA) and VWR (Radnor, PA, USA).

### 4.2. Protein Preparation

Human cardiac myosin constructs contain 1–843 residues and a FLAG affinity tag at the C-terminus. Corresponding adenoviruses, encoded the wild-type myosin construct and I303V:I313V myosin mutant, were purchased from Vector Biolabs (Malvern, PA, USA). We amplified adenoviruses using HEK293 cells (ATCC CRL-1573), and purified them using CsCl gradient centrifugation. Human cardiac myosin constructs were expressed in C_2_C_12_ (ATCC CRL-1722) mouse myoblast cells. We grew C_2_C_12_ cells to a 95% confluence on 150 mm diameter tissue-culture dishes and infected cells with the optimum dosage of virus determined previously by a viral titration assay. Cells were collected seven days post-infection and the myosin construct was extracted and purified. We used anti-FLAG magnetic beads (Sigma-Aldrich, Milwaukee, WI, USA) and 3x FLAG peptide (ApexBio, Houston, TX, USA) in the myosin purification procedure. Myosin purity was examined using the Coomassie-stained SDS-PAGE. Myosin concentration was determined by measuring UV absorbance at 280 nm using extinction coefficient ε_280nm_ = 93,170 M^−1^cm^−1^, calculated using the ProtParam tool of ExPASy web server.

Actin was prepared from New Zealand white junior doe (3–4 months of age) leg and back muscles [17,18,19]. The sex of the animal does not produce any variation in prepared protein [20]. We labeled F-actin with pyrene iodoacetamide (Life Technologies Corporation, Grand Island, NY), the molar ratio 4:1 (label:actin). Labeled actin was stabilized with phalloidin at the molar ratio of 1:1 and dialyzed against the experimental buffer at T = 4 °C. Concentration of unlabeled G-actin was determined using the extinction coefficient ε_290nm_ = 0.63 (mg/mL)^−1^cm^−1^ [21]. Concentration of labeled G-actin and labeling efficiency were determined using the following expressions: [G-actin] = (A_290nm_ − (A_344nm_·0.127))/26,600 M^−1^ and [pyrene] = A_344nm_/22,000 M^−1^ [22]. In this work, actin labeling efficiency was 40–80%. Actin produced from three animals was used in the reported experiments. The experimental buffer contained 20 mM MOPS (3-[*N*-morpholino]propanesulfonic acid) pH 7.3, 50 mM KCl, 3 mM MgCl_2_. All reported concentrations are final concentrations.

### 4.3. Acquisition of Fluorescent Transients

Transient fluorescence of pyrene labeled actomyosin was measured using Bio-Logic SFM-300 stopped flow transient fluorimeter (Bio-Logic Science Instruments SAS, Claix, France) equipped with the FC-15 cuvette. Actomyosin of the concentration 0.5 µM–1 µM was used in all transient kinetics experiments. To measure the rate of the ATP-induced actomyosin dissociation, we rapidly mixed actomyosin and ATP solution of the concentration 15 µM–600 µM. To measure the rate of the ADP inhibition of the ATP-induced actomyosin dissociation, we rapidly mixed actomyosin with the premixed ATP and ADP solution; the concentration of ATP was 600 µM and the concentration of ADP was 10 µM–200 µM. The fluorescence of pyrene actin was excited at 365 nm, and we used a 420 nm cutoff filter to detect the fluorescence. We acquired and averaged up to twenty transients to improve the signal-to-noise ratio. A total of 8000 data points were acquired in each experiment. All experiments were performed at T = 20 °C. All reported experiments, ATP-induced actomyosin dissociation, and ADP release from actomyosin were performed with myosin constructs from at least three independent cell growths and protein preparations.

### 4.4. Analysis of Fluorescence Transients

**Figure 4:**
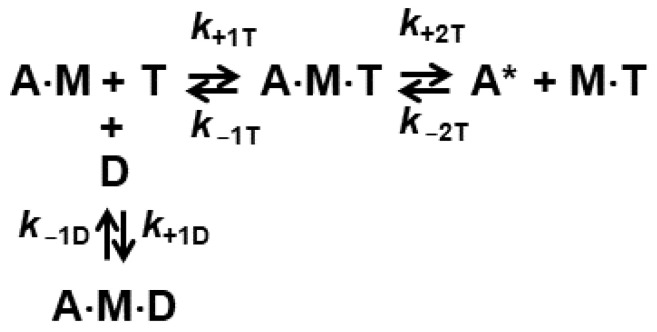
Inhibition of the ATP-induced actomyosin dissociation with ADP. A = pyrene-labeled actin, M = myosin, T = ATP. A* = actin with the high pyrene fluorescence.

Obtained transients were initially fitted with the one-exponential function S(*t*) = S_o_ + A·exp(−*k*_obs_·(*t* − *t*_0_)), where S(*t*) is the observed signal at the time t, A is the signal amplitude, t_0_ is the time before the flow stops, and *k*_obs_ is the observed rate constant. The one-exponential transient means rapid equilibrium of step 1 (Figure 2), or steps 1 and D (Figure 4), followed by the exponential growth of pyrene-actin fluorescence, reflecting actomyosin dissociation. For the WT myosin construct, we obtained a good one-exponential fit of transients of ATP-induced actomyosin dissociation. The fit of transients of the mutant was better with the double-exponential function, with the fast and slow components. The slow component did not show any dependence on ATP concentration, and the amplitude of the component was virtually constant (13 ± 3%) in different protein preparations. The kinetic rate of actomyosin dissociation of the slow component is 76.5 ± 20.3 s^−1^ at all used concentrations of ATP. Most likely, this component reflects a small amount of somewhat misfolded myosin mutant, able to bind actin strongly, but binding ATP slower than the rest of the myosin construct. Therefore, to account for this component and remove it from our consideration, we used the determined amplitude and rate of the component and subtracted the corresponding single-exponential function from obtained transients of the mutant before all our fits.

The dependence of the observed rates *k*_obs_ on the ATP concentration was fitted by a hyperbola, *k*_obs_ = *k*_+2T_·[ATP]/((1/*K*_1T_) + [ATP]), where *k*_+2T_ is the maximum rate (the horizontal asymptote), and *K*_1T_ is the equilibrium association constant of the collision complex formation. The ATP dependence of the observed rates at small concentrations of ATP was fitted with a straight line to determine the bimolecular rate *K*_1T_*k*_+2T_.

**Figure 5:**
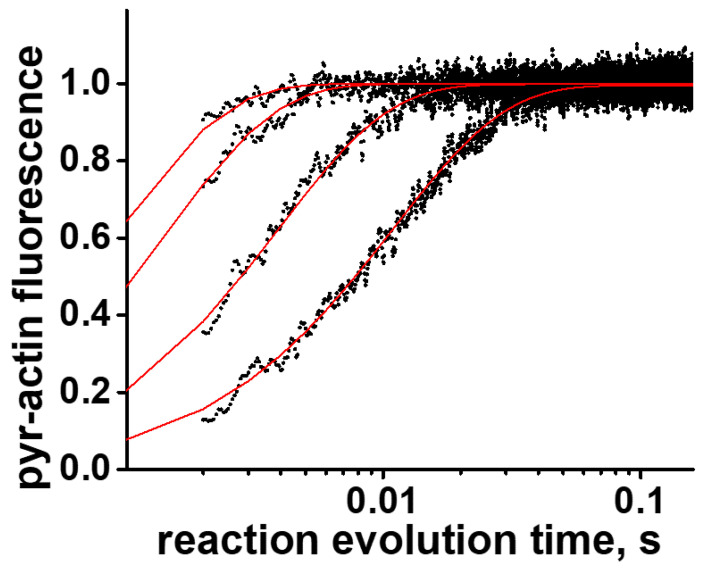
Example of fitted transients of ATP-induced actomyosin dissociation (reaction scheme Figure 2) to the numerical solution of the system of differential equations (Supplementary Equation (S1)). Transients, obtained for the same actomyosin preparation at different concentrations of ATP are fitted globally. The fitted parameters are *k*_+1T_, *k*_−1T_, *k*_+2T_, *k*_−2T_. Black dots, experimental data. Red lines, global fit. Actomyosin concentration is 1 µM, ATP concentrations are 30 µM, 90 µM, 300 µM, 600 µM.

We also used the numerical solution of the system of ordinary differential equations, which correspond to the reaction scheme shown in Figure 2 to fit transients obtained in the ATP-induced actomyosin dissociation experiment. The reaction rates, obtained in fits with exponentials and with the solution of differential equations, were in excellent agreement (see Results). In all our fits, transients obtained for the same actomyosin preparation were fitted globally, assuming the same intensity of pyrene-actin fluorescence at the mixing point and in the end of the reaction, because of complete dissociation of actomyosin (Figure 5).

**Figure 6:**
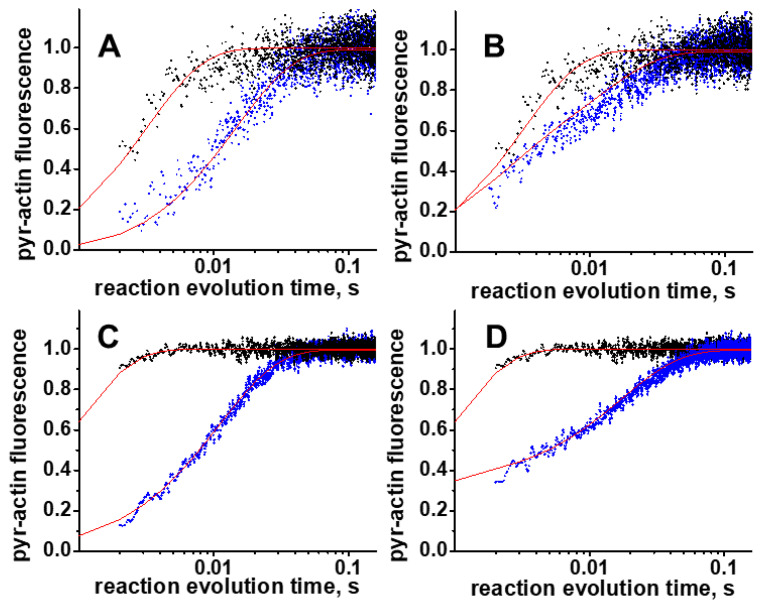
Typical transients, obtained in the study. (**A**,**B**), WT, and (**C**,**D**) mutant myosin construct. (**A**,**C**), the ATP-induced actomyosin dissociation experiment. Pyrene-labeled actomyosin is rapidly mixed with ATP ([ATP] = 30 μM, blue dots, 600 μM, black dots). (**B**,**D**), the ADP inhibition experiment. Pyrene-labeled actomyosin is rapidly mixed with the premixed ATP and ADP ([ATP] = 600 µM, [ADP] = 0 µM, upper trace, black dots, and [ADP] = 100 µM, lower trace, blue dots). Normalized pyrene actin fluorescence reflects actomyosin dissociation. Red traces, fits to the numerical solution of the system of differential equations (Supplementary Equations (S1) and (S2)). For the ATP-induced actomyosin dissociation experiment, the fitted parameters are *k*_+1T_, *k*_−1T_, *k*_+2T_, *k*_−2T_. For the ADP inhibition experiment, the fitted parameters are *k*_+1D_, *k*_+1D_. A different signal-to-noise ratio reflects different labeling efficiency of actin.

In the ADP inhibition experiment, transients can be best fitted with a two-exponential function. The rate constant of the fast exponential presumably reflects the ATP-induced actomyosin dissociation, and the rate constant of the slow exponential is governed by the ADP release from actomyosin. The fit with the two-exponential function confirms that ADP is not in fast equilibrium with actomyosin, and the reaction must be treated as two parallel reactions. We fitted obtained transients with the numerical solution of the ordinary differential equations, corresponding to the reaction scheme in Figure 4, assuming that ADP and actomyosin are not in the fast equilibrium (Figure 6). All exponential data fits were conducted with Origin (OriginLab Corp, Northampton, MA, USA). The system of differential equations was solved numerically using Wolfram Mathematica’s built-in symbol NDSolve. The numerical solution was fitted to the experimental data using the built-in symbol NMinimize, which searches for a global minimum. We obtained parameters *k*_+1T_, *k*_−1T_, *k*_+2T_, *k*_−2T_ from fits of the data of the ATP-induced actomyosin dissociation experiment. Obtained rate constants were averaged, and mean values were used in the global fits of the transients of the ADP inhibition experiments to determine fitted parameters *k*_+1D_ and *k*_+1D_. To confirm the robustness of the fit, we also fitted the transients of the ADP inhibition experiment using determined kinetic rate constants for ATP and ADP binding as constants, and varying kinetic rate constants of actomyosin dissociation. These fits were of the same quality, and kinetic rate constants of actomyosin dissociation were statistically similar to the rate constants, obtained in the fit of the transients of the ATP-induced actomyosin dissociation experiment. Obtained kinetic rate constants (fitted parameters) were averaged and reported in Table 1 as a mean ± standard deviation. Wolfram Mathematica scripts are provided in the Supplementary Material file.

### 4.5. Statistical Analysis

We used two-sample t-test analysis to examine if the kinetic constants of the WT and the mutant are statistically different. The *p*-value was calculated to determine if the null hypothesis (equal kinetic constants for the WT and mutant myosin constructs) or the alternative hypothesis (not equal kinetic constants for the WT and mutant myosin constructs) is true. We assumed that the *p*-value less than the significance level of 0.05 indicates that the null hypothesis can be rejected. The statistical analysis was performed using the statistics package integrated into Origin (OriginLab Corp, Northampton, MA, USA) software, assuming that the kinetic constants are independent and normally distributed with unequal variances.

## Figures and Tables

**Table 1:** Obtained kinetic rate constants. Results of the fit of the transients with the numerical solution of the system of differential equations (Supplementary Equations (S1) and (S2)), mean ± SD. The rate constant *K*_1T_*k*_+2T_ was obtained from the fit shown in Figure 3A. Data are averages of three independent protein preparations each for WT and mutant constructs.

	WT ^(a)^	I303V:I313V
**Actomyosin dissociation**		
*k*_+1T_, µM^−1^s^−1^	7.42 ± 1.35	12.83 ± 1.61
*k*_−1T_, s^−1^	661.0 ± 72.9	5877.84 ± 1025.1
*k*_+2T_, s^−1^	406.2 ± 14.1	2307.6 ± 389.6
*k*_−2T_, s^−1^	3.6 ± 2.5	0.9 ± 0.5
*K*_1T_*k*_+2T_, µM^−1^s^−1^	2.12 ± 0.21	3.54 ± 0.07
**Actomyosin collision complex dissociation**		
1/*K*_1T_, µM	89.6 ± 6.5	461.7 ± 86.7
**ADP binding and release from actomyosin**		
*k*_+1D_, µM^−1^s^−1^	26.6 ± 8.1	41.6 ± 3.1
*k*_−1D_, s^−1^	199.6 ± 51.8	175.7 ± 8.7
**ADP dissociation from actomyosin**		
*K*_1D_, µM	7.5 ± 3.0	4.2 ± 0.4

^(a)^ Data from [4].

## Data Availability

Raw data generated during the current study are available from the corresponding author on reasonable request. All analyzed data are included in this published article.

## Supplementary Materials

The following supporting information can be downloaded at: https://www.mdpi.com/article/10.3390/ijms23031220/s1, Equations (S1) and (S2) and Wolfram Mathematica scripts.
