# Supplementary material for: The Local Environment of Loop Switch 1 Modulates the Rate of ATP-Induced Dissociation of Human Cardiac Actomyosin
Source: Int J Mol Sci. 2022 Jan 22;23(3):1220. doi: 10.3390/ijms23031220 (PMC8835826; doi:10.3390/ijms23031220)
Supplement: Supplementary file 1 [file ijms-23-01220-s001.zip › ijms-1538840-supplementary.pdf]

Supplementary Material

The local environment of loop switch 1 modulates the rate of ATP-induced dissociation of human cardiac actomyosin

Akhil Gargey[1,2], Yuri E. Nesmelov[1,*]

[1]Department of Physics and Optical Science, University of North Carolina Charlotte, Charlotte NC 28223
[2]Department of Biological Science, University of North Carolina Charlotte, Charlotte NC 28223

Differential equations were solved numerically using Wolfram Mathematica built-in symbol NDSolve. The solution was fitted to the experimental data using the built-in symbol NMinimize, which searches for a global minimum. The maximum number of iterations was usually set to 100. If minimization was not completed, a maximum of 200 iterations was used. We first fitted transients from the experiment with [ADP] = 0 (Figure 2) to determine reaction rate constants k_{+1T}, k_{-1T}, k_{+2T}, k_{-2T}. Transients obtained from the same myosin construct preparation were fitted globally. We used obtained reaction rate constants in the fit of transients, obtained in experiments with [ADP] ≠ 0. All transients obtained in reactions using myosin from the same preparation were fitted globally for all used ADP concentrations. Reaction rate constants determined from the fits for different preparations of the same myosin construct were averaged and reported as mean ± standard deviation.

1. Differential equations corresponding to the reaction shown in Scheme 1.

$$\frac{dAM}{dt} = -k_{+1T}AM \cdot T + k_{-1T}AMT$$
$$\frac{dAMT}{dt} = k_{+1T}AM \cdot T - k_{-1T}AMT - k_{+2T}AMT + k_{-2T}A \cdot MT$$
$$\frac{dMT}{dt} = k_{+2T}AMT - k_{-2T}A \cdot MT$$
$$\frac{dA}{dt} = k_{+2T}AMT - k_{-2T}A \cdot MT$$

Eq. S1

2. Wolfram Mathematica script to solve the system of differential equations Eq. S1 numerically and fit obtained transient to the numerical solution. Input: data (ASCII file with the experimental data). Output: rate constants k_{+1T}, k_{-1T}, k_{+2T}, k_{-2T}.

```
sse[kp1T_?NumberQ,km1T_?NumberQ,kp2T_?NumberQ,km2T_?NumberQ]:=Block[{sol},sol=NDSolve[{
am'[x]==-kp1T*am[x]*T+km1T*amt[x],
amt'[x]==kp1T*am[x]*T-(km1T+kp2T)*amt[x]+km2T*a[x]*mt[x],
a'[x]==kp2T*amt[x]-km2T*a[x]*mt[x],
mt'[x]==kp2T*amt[x]-km2T*a[x]*mt[x],
```

```
am[0]==0.5, amt[0]==0,a[0]==0,mt[0]==0},{a},{x,0,0.8}][[1]];Plus@@Apply[(a[#1]-#2)^2&,data,{1}]/.sol]
NMinimize[{sse[kp1T,km1T,kp2T,km2T],0.5<=kp1T<=80,100<=km1T<=3500,50<=kp2T<=1600,0.001<=km2T<=50},{
{kp1T,1,15},{km1T,300,500},{kp2T,500,600},{km2T,1,2}}]
```

3. Differential equations corresponding to the reaction shown in Figure 3.

$$\frac{dAM}{dt} = -k_{+1T}AM \cdot T + k_{-1T}AMT - k_{+1D}AM \cdot D + k_{-1D}AMD \quad \text{Eq. S2}$$
$$\frac{dAMT}{dt} = k_{+1T}AM \cdot T - (k_{-1T} + k_{+2T})AMT + k_{-2T}AM \cdot T$$
$$\frac{dA}{dt} = k_{+2T}AMT - k_{-2T}A \cdot MT$$
$$\frac{dMT}{dt} = k_{+2T}AMT - k_{-2T}A \cdot MT$$
$$\frac{dAMD}{dt} = k_{+1D}AM \cdot D - k_{-1D}AMD$$
$$\frac{dD}{dt} = -k_{+1D}AM \cdot D + k_{-1D}AMD$$

4. Wolfram Mathematica script to solve the system of differential equations Eq. S2 numerically and fit the obtained transient to the numerical solution. Input: data (ASCII file with the experimental data), rate constants k_{+1T}, k_{-1T}, k_{+2T}, k_{-2T}, determined in the previous fit. Output: rate constants k_{+1D}, k_{-1D}.

```
sse[kp1D_?NumberQ,km1D_?NumberQ]:=Block[{sol},sol=NDSolve[{
    am'[x]==-kp1T*am[x]*T+km1T*amt[x]-kp1D*am[x]*d[x]+km1D*amd[x],
    amt'[x]==kp1T*am[x]*T-(km1T+kp2T)*amt[x]+km2T*a[x]*mt[x],
    a'[x]==kp2T*amt[x]-km2T*a[x]*mt[x],
    mt'[x]==kp2T*amt[x]-km2T*a[x]*mt[x],
    amd'[x]==kp1D*am[x]*d[x]-km1D*amd[x],
    d'[x]==-kp1D*am[x]*d[x]+km1D*amd[x],
    am[0]==0.5, amt[0]==0,a[0]==0,mt[0]==0,d[0]==ADP,amd[0]==0},{a},{x,0,0.8},][[1]];Plus@@Apply[(a[#1]-
#2)^2&,data,{1}]/.sol]
NMinimize[{sse[kp1D,km1D],1<=kp1D<=200,10<=km1D<=800},{{kp1D,1,10},{km1D,100,200}}]
```
